# Polio by the Numbers—A Global Perspective^[Author-notes jiac130-FM1]^

**DOI:** 10.1093/infdis/jiac130

**Published:** 2022-04-13

**Authors:** Kamran Badizadegan, Dominika A Kalkowska, Kimberly M Thompson

**Affiliations:** Kid Risk, Inc, Orlando, Florida, USA; Kid Risk, Inc, Orlando, Florida, USA; Kid Risk, Inc, Orlando, Florida, USA

**Keywords:** polio, vaccines, modeling, disease eradication, prevention

## Abstract

**Background:**

Investments in national immunization programs and the Global Polio Eradication Initiative (GPEI) have resulted in substantial reductions in paralytic polio worldwide. However, cases prevented because of investments in immunization programs and GPEI remain incompletely characterized.

**Methods:**

Using a global model that integrates polio transmission, immunity, and vaccine dynamics, we provide estimates of polio incidence and numbers of paralytic cases prevented. We compare the results with reported cases and estimates historically published by the World Health Organization.

**Results:**

We estimate that the existence and use of polio vaccines prevented 5 million cases of paralytic polio for 1960–1987 and 24 million cases worldwide for 1988–2021 compared to a counterfactual world with no polio vaccines. Since the 1988 resolution to eradicate polio, our estimates suggest GPEI prevented 2.5–6 million cases of paralytic polio compared to counterfactual worlds without GPEI that assume different levels of intensity of polio vaccine use in routine immunization programs.

**Conclusions:**

Analysis of historical cases provides important context for understanding and communicating the benefits of investments made in polio eradication. Prospective studies will need to explore the expected benefits of future investments, the outcomes of which will depend on whether and when polio is globally eradicated.

## Teaser

Since 1960, polio immunization has prevented nearly 30 million cases of paralysis compared to a world with no polio vaccines. We estimate that the Global Polio Eradication Initiative has decreased polio cases by 2.5–6 million since it began in 1988.

**(See the Editorial Commentary by Bandyopadhyay and Orenstein, on pages 1301–3.)**

Following the introduction of polio vaccines in the 1950s, many countries stopped poliovirus transmission nationally within a few decades (eg, the United States, reviewed in [1]). In 1985, the Pan American Health Organization launched a successful campaign to end polio in the western hemisphere [2]. Demonstration of regional polio elimination in the Americas helped to motivate the 1988 World Health Assembly commitment to the global eradication of poliomyelitis.

Since 1988, the Global Polio Eradication Initiative (GPEI) has coordinated efforts to stop poliovirus transmission in 105 countries that required external financial support [3, 4]. As of 2022, GPEI has received cumulative financing of approximately $20 billion (US$ 2021), with an annual budget of around $1 billion [5]. The eradication of indigenous transmission of wild polioviruses (WPVs) was certified for type 2 in 2015 [6] and for type 3 in 2019 [7]. Type 1 transmission remains limited to Pakistan and Afghanistan [8, 9]. However, the polio endgame is complicated, particularly due to vaccine-derived polioviruses (VDPVs) [8, 9]. Following the global eradication of WPV types 2 and 3, circulating VDPVs (cVDPVs) became the only reported cases for these types, and in 2021, for the first time, reported cases of type 1 cVDPVs exceeded those caused by WPVs [10, 11].

Public health authorities and health economists typically value the benefits of an intervention based on the number of bad outcomes avoided, such as lives saved, or morbidity prevented. This involves a model-based process to characterize events that did not happen. The World Health Organization (WHO) publishes annual paralytic polio cases since 1980 based on aggregation of national surveillance data including reported cases of WPVs and cVDPVs [12, 13]. Surveillance data are often imperfect, and estimated numbers of polio cases or cases prevented are not readily available. Prior studies characterized financial costs and benefits of polio immunization efforts using different assumptions and framing, some including estimates of annual and/or cumulative polio cases and cases prevented [4, 14 (review)]. Overall, estimates of polio cases prevented globally due to polio vaccine use and GPEI resources remain poorly synthesized.

While WHO historically published estimates based on simple linear models of polio incidence and cases prevented, these stopped in 1990s. Simple calculations can provide useful but occasionally misleading assessments due to simplistic assumptions and inclusion/exclusion of benefits attributable to intervention(s). Well-calibrated global disease models can provide a basis for assessment of the simple approach and put the results in context. We have developed and maintain the only global model that integrates poliovirus transmission, immunity, vaccine dynamics, and the economics of interventions [14, 15]. Many of our modeling studies retrieved reported and projected polio cases, considered these during model calibration, and provided estimates of cases and cases prevented as a function of time (eg, [3, 4, 15]). However, these studies focused primarily on policy and economic analyses, and not on reporting specific numbers of cases and cases prevented. Here, we provide a high-level summary of the reported and estimated annual cases from our integrated model [15], expand on key concepts related to different estimates of polio prevented by different interventions, and compare our estimates with those published by WHO. While we focus on paralytic cases, we emphasize that polio historically caused mortality at an estimated rate of 10% of paralytic cases [16], although case-fatality rates vary by age and country [17].

## METHODS

We reviewed the literature to identify global reported cases of polio by year, estimates of polio cases, and estimates of polio cases prevented. The search included WHO electronic archives of publications.

We applied our integrated global polio transmission and oral poliovirus vaccine (OPV) evolution model [4, 15] to demonstrate how different assumptions change the estimates of cases for different interventions. The model divides the global population estimated in the United Nations World Population Prospects (2019 Revision) [18] for 200 countries into subpopulations of approximately 10 million people that abstractly reflect actual populations, and organizes them by World Bank income level and the current polio vaccine use according to routine immunization (RI) schedules using OPV and inactivated poliovirus vaccine (IPV) [15]. The model tracks dynamics of infections and immunity for all 3 types of polio using 720 subpopulations, 7 age groups, 8 immunity states, 5 stages for waning, and a 20-stage process for evolution of OPV to cVDPVs [15]. The RI schedule is a function of the population block and consists of OPV + IPV with an IPV dose given simultaneously with the third OPV dose, or sequential IPV/OPV schedule that gives IPV first followed by OPV, or IPV only [15]. We characterize the time series of RI coverage using WHO/UNICEF Estimates of National Immunization Coverage for diphtheria–tetanus–pertussis 3-dose (DTP3) for each country for each year [19]. These correlate with coverage of 3 doses of polio vaccine and provide RI coverage estimates that are not affected by polio supplementary immunization activities (SIAs), for which the model uses separate subpopulation- and time-specific inputs [15]. We updated all model immunization inputs through the end of 2021 to simulate the global poliovirus transmission with historical use of all polio vaccines (in RI and SIAs), and to reflect the disruptions in immunization activities that occurred due to coronavirus disease 2019 (COVID-19) [20]. We define the baseline model of historical global experience as the reference case (RC).

We also estimate annual polio incidence using a simple model similar to the one used historically by WHO (see discussion in [15]). We sum over the same 200 countries as in the global transmission model (*c*) to obtain yearly global incidence, INC(yr), yr = 1980,..., 2021. For consistency with the RC, we use the same inputs for RI coverage and surviving infants (*SI*) for each year. We then use the formula:

$$INC(yr)=\;\overset{200}{\underset{c=1}{\sum }}\;SI_{c}(yr)\times (1-tr\times DTP3_{c}(yr))\times PIR$$

where *tr* is the average take rate for OPV and *PIR* is the paralysis-to-infection ratio (PIR). The average *tr* represents a substantial simplification relative to the global transmission model, which uses subpopulation-specific and type-specific take rates for different formulations of polio vaccines [15].

As demonstrated previously [15], we consider different input assumptions to provide insights about the sensitivity of the simple model to these inputs. We consider 2 average take rates (*tr* = 95% or 80%), either a fixed average paralysis-to-infection ratio (aPIR) of 1/200, or serotype-specific paralysis-to-infection ratio (sPIR) of 1/200, 1/2000, and 1/1000 for poliovirus serotypes 1, 2, and 3, respectively, and consider the effect of counting or not counting the contribution of cases from countries that achieved WPV elimination (+elim or −elim). As before [15], we show 4 of 8 possible permutations including: aPIR+elim+95%, sPIR+elim+95%, aPIR+ elim+80%, and aPIR−elim+95%. Table 1 summarizes the differences in the key inputs for these analyses.

**Table 1.** Summary of Key Model Inputs that Differ for These Analyses [15]

Scenario	Model Inputs						
	Take Rate, %			Paralysis-to-Infection Ratio			Elimination Time
	Type 1	Type 2	Type 3	Type 1	Type 2	Type 3	
Global model, RC	35–95^a^	60–98^a^	27–90^a^	1/200	1/2000	1/1000	Simulated
aPIR+elim+95%	95	95	95	1/200	1/200	1/200	Same as RC^b^
aPIR+elim+80%	80	80	80	1/200	1/200	1/200	Same as RC^b^
sPIR+elim+95%	95	95	95	1/200	1/2000	1/1000	Same as RC^b^
aPIR-elim+95%	95	95	95	1/200	1/200	1/200	None^c^

Abbreviations: aPIR, average paralysis-to-infection ratio; elim, elimination; RC, reference case; sPIR, serotype-specific paralysis-to-infection ratio.

a As indicated in the text, the global model uses subpopulation-specific and vaccine-specific take rates that vary within the ranges indicated. See [16] for details.

b In these 3 scenarios, elimination time is set to be equal to the simulated elimination time in the RC to ensure no counting of cases after elimination.

c This scenario implicitly assumes that cases accumulate indefinitely, even after elimination.

To characterize cases prevented, we compare what happens with an intervention to what happens without it, and one of these necessarily represents a counterfactual scenario (ie, something that did not happen). Our focus on global estimates limits the analytical framing choices to different time periods and different interventions. We demonstrate different choices for global impacts of (1) introduction of polio vaccines or (2) actions attributable to GPEI. As demonstrated in prior economic analyses of GPEI [3, 4], most developed countries had already committed to national and regional elimination of polio prior to 1988. Therefore, as an intervention, the time horizon of GPEI starts in 1988 and includes only the countries that received direct support [3, 4]. Thus, for the counterfactual scenarios we consider (1) the world with no polio vaccine (no vaccine) and (2) the world with polio vaccines but no GPEI (no GPEI). Further, we consider 3 no GPEI scenario options by varying the intensity of polio vaccine use in RI [21]. Specifically, we assume that countries would have continued using trivalent OPV (tOPV) for 3 different trajectories of RI coverage between 1988 and 2021:

- Flat (constant) coverage equal to the 1988 RI estimate, or
- Increasing RI coverage that follows the historically reported time series of RI coverage, or
- Increasing RI coverage that linearly increases between 1988 and 2021 to the mid-level between reported 1988 and 2019.

The no GPEI scenarios include no planned, preventive SIAs, and we assume regular WPV importations throughout the time horizon into subpopulations that achieved local WPV elimination. Virus is imported into these subpopulations from countries or subpopulations that remain endemic (ie, those yet to achieve national polio elimination). Because of importation threats, we assume that the blocks using IPV-only schedules in the RC [15] would use an IPV/OPV sequential schedule for RI (instead of IPV-only) to minimize vaccine-associated paralytic polio, while continuing to benefit from high population immunity from the intestinal immunity induced by OPV [22].

## RESULTS

### Reported Paralytic Polio

**Figure 1.**
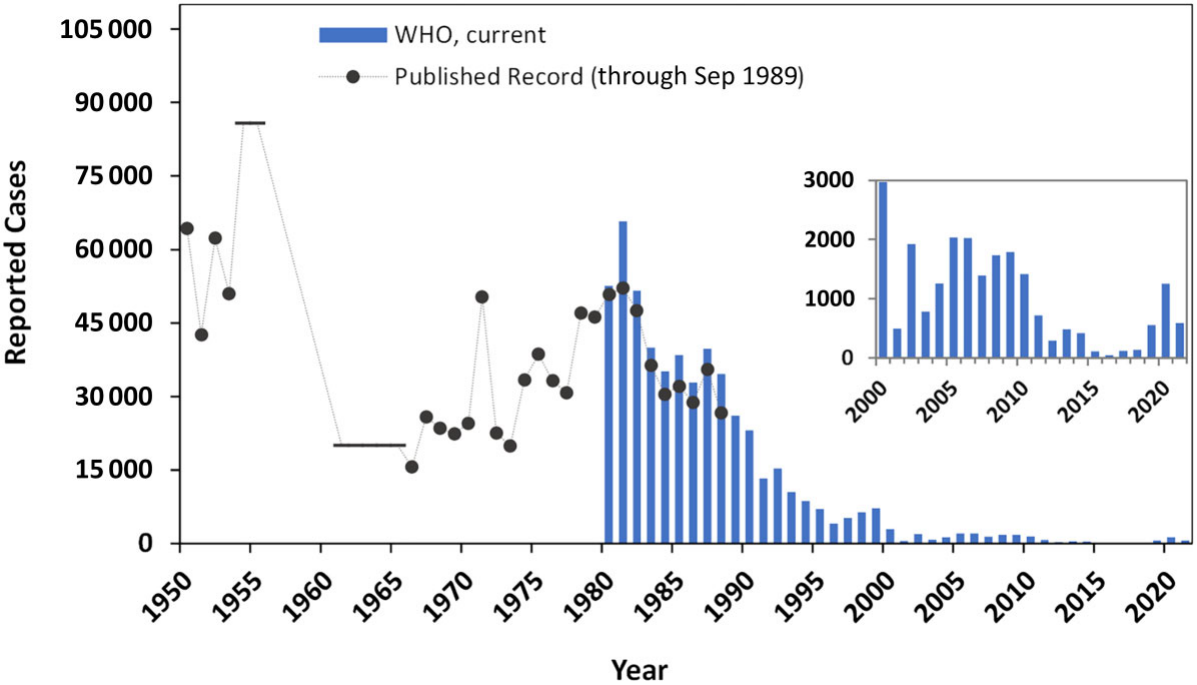
The total number of globally reported cases from 1950 through 2021. Blue bars show reported cases published by the World Health Organization (WHO) from 1980 through 29 December 2021 [12, 13]. Solid black circles are the most up-to-date annual reported cases in WHO published reports as of mid-1989 [23–30], representing the best available data at the time of 1988 World Health Assembly resolution to eradicate polio. The solid horizontal bars covering the periods 1954–1955 and 1961–1965 are published averages for these periods [24]. Inset highlights the period between 2000 and 2021 at a magnified vertical axis scale.

Figure 1 shows the total number of reported cases starting from 1950, shortly before vaccines became available. Reported cases theoretically represent the sum from all countries, but countries with limited healthcare resources or poor surveillance missed cases that were either not diagnosed or not reported. Overall, the available record between 1950 and 1980 remains incomplete and characterized by periodic WHO revisions. The solid circles in Figure 1 represent the most up-to-date data for each year as of mid-1989 [23–30], representing contemporaneous data at the time of the 1988 World Health Assembly resolution to eradicate polio. The solid horizontal bars between 1954–1955 and 1961–1965 are published averages [24]. We were unable to locate published annual data for these periods, or any published global data for the period 1956–1960. Reported cases between 1950 and 1953 in some but not all countries distinguish “paralytic cases” from “all forms.” We recorded paralytic cases when available and assume single numbers from other countries represent paralytic cases.

The annual data since 1980 currently reported by WHO [12, 13] appear in blue bars in Figure 1 and include both WPVs and cVDPVs of all types. The discrepancy between blue bars and circles highlights uncertainty about reported cases. Retroactive updates by WHO (often in the positive direction of additional cases with increased reporting) are not uncommon, and we assumed that later reports supersede earlier reports for any given year.

### Estimates of Total Paralytic Polio

**Figure 2.**
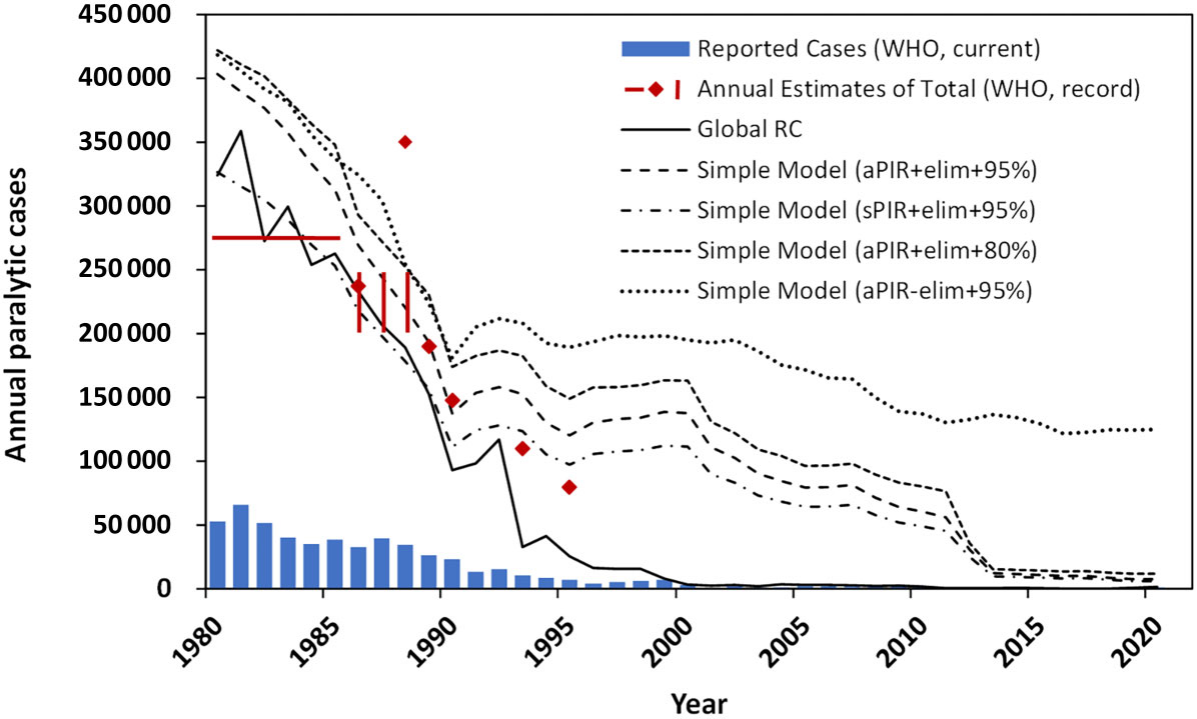
The total number of globally reported cases from 1980 through 2021. Blue bars show reported cases by WHO from 1980 through 29 December 2021 [12, 13]. Red diamonds show published point estimates [32–36]. Solid horizonal red line represents published average point estimate for the years 1980 to 1985 [29]. Solid red vertical bars represent published range estimate for the years 1986 to 1988 [31]. Solid black curve shows annual paralytic cases of the integrated global model reference case (global RC). Dashed and dotted curves show annual global paralytic cases estimated using simple models for parameters indicated in the figure legend. Abbreviations: aPIR, average paralysis-to-infection ratio; elim, elimination; RC, reference case; sPIR, serotype-specific paralysis-to-infection ratio; WHO, World Health Organization.

Recognizing underreporting as a significant data quality issue, WHO used a simple linear model in the 1980s to estimate the total number of paralytic cases [29, 31–36]. In these estimates (Figure 2, red diamond symbols and lines), WHO made simplifying assumptions, including average PIR (aPIR) of 1 in 200 regardless of poliovirus type, fixed incidence rate of 5 per 1000 newborns, RI with *tr* = 95%, and vaccine coverage of zero for countries with no available data. Figure 2 also includes annual paralytic cases from our integrated global RC (solid black curve). The RC accounts for all activities of GPEI, including SIAs. Comparison of various estimates with reported cases (blue bars) highlight the gap due to underreporting.

Not surprisingly, published WHO estimates (red diamond symbols) largely overlap with the simple linear models (dashed and dotted curves). These point estimates tend to overestimate the total paralytic cases compared to the RC, demonstrated by the divergence of the solid line from other lines beginning in the 1988–1990 timeframe, when polio eradication activities increasingly contributed to reducing the global burden of polio. The difference between the RC and simple models becomes so large after the mid-1990s that simple models do not reliably estimate the global burden of disease, or the number of cases prevented (see below).

**Figure 3.**
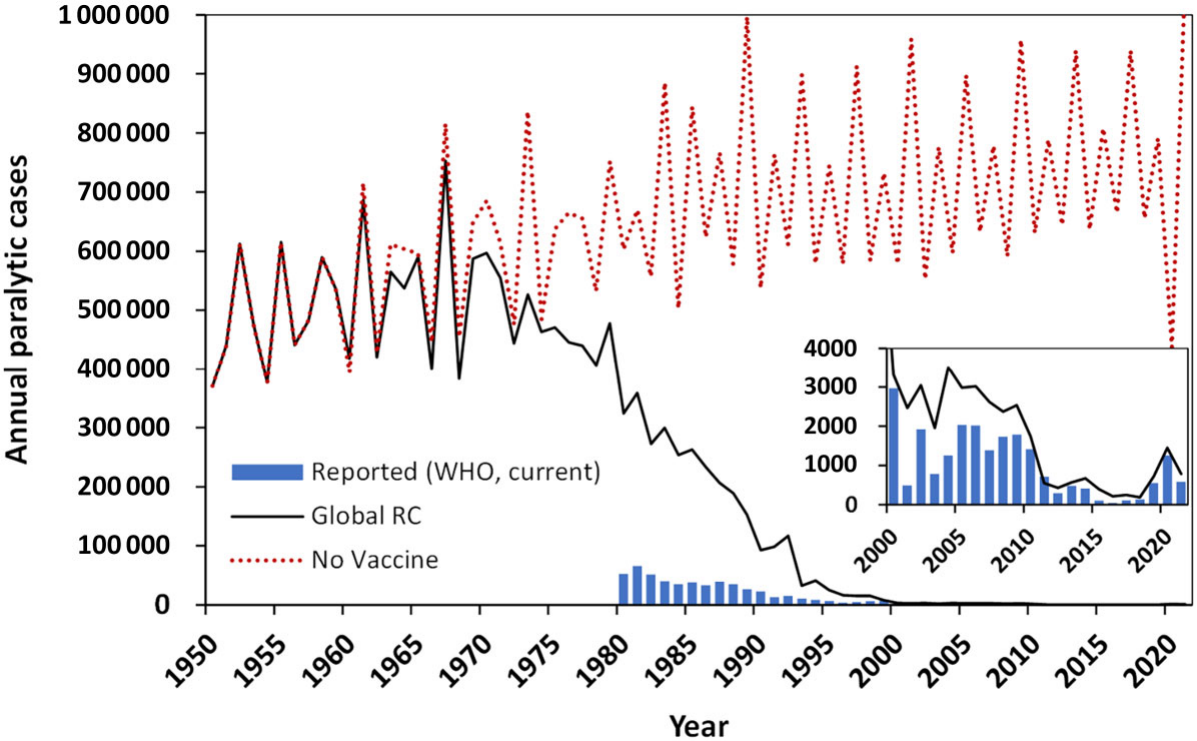
Estimated total annual paralytic polio cases since 1950 with GPEI (solid black curve) and without poliovirus vaccines (dotted red curve). For reference, blue bars show reported cases of paralytic polio through 29 December 2021 [12, 13]. The inset shows the period 2000 to 2021 with a magnified vertical axis to make visible the reported and modeled cases of paralytic polio in the past 2 decades. Abbreviations: GPEI, Global Polio Eradication Initiative; RC, reference case; WHO, World Health Organization.

In Figure 3, we extend the RC to show estimated cases for 1950 to 2021 (solid black curve), and the estimated annual paralytic cases without the introduction of poliovirus vaccines (no vaccine, dotted red curve). The RC introduces polio vaccines starting in 1960 to simulate the global uptake timeline. With increasing adoption of vaccines over time, the 2 curves diverge substantially. In the absence of vaccines, expected polio cases show an epidemic behavior that oscillates around the size of the cohort of surviving infants, whereas the RC that includes all national and international polio immunization activities, results in near elimination of polio worldwide. The magnified inset in Figure 3 highlights the continuation of global eradication efforts, because the current burden of polio becomes virtually invisible compared to the hypothetical background of a world without polio vaccines.

### Estimates of Paralytic Polio Prevented

**Figure 4.**
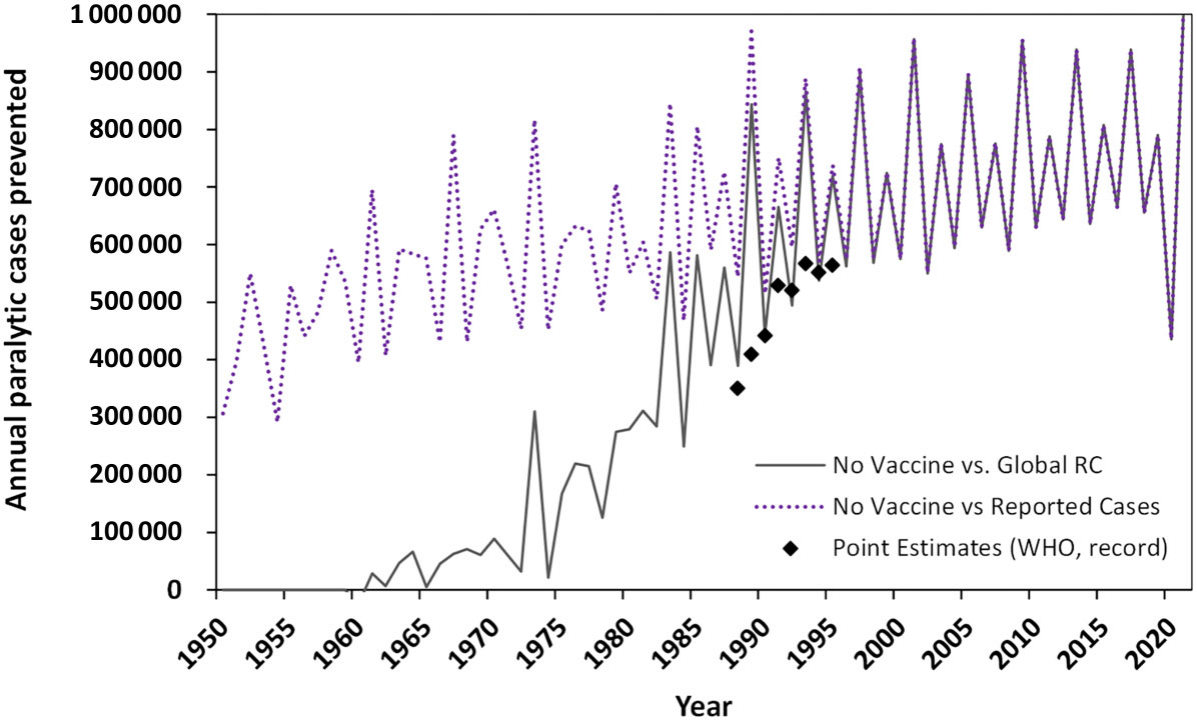
Estimated total annual paralytic polio cases prevented since 1950 using the no vaccine scenario compared to either the global RC (solid black curve) or the reported cases (dotted purple curve). Black diamonds show WHO published point estimates of the paralytic polio cases prevented for the years 1988 to 1995 [31, 32, 37–42–38–42]. Abbreviations: RC, reference case; WHO, World Health Organization.

Figure 4 shows 2 different ways to estimate annual paralytic cases prevented. First, the no vaccine vs global RC (solid black) curve in Figure 4 subtracts the number of cases of the Global RC (solid black curve in Figure 3) from the counterfactual no vaccine scenario (red dotted curve in Figure 3). In the purple dotted curve, Figure 4 also shows the result of subtracting the reported cases (blue bars) from the no vaccine scenario, which substantially exaggerates the benefits of polio vaccinations until the 1980s. Although the purple dots may easily be estimated, they fail to incorporate the well-known significant underreporting of paralytic polio (Figure 2). Figure 4 also shows published WHO estimates of cases prevented in black diamond symbols. We could not identify a published source for the methodology that WHO used to obtain its global estimates, and we only found estimates for 1988 to 1995 [31, 32, 37–42].

**Figure 5.**
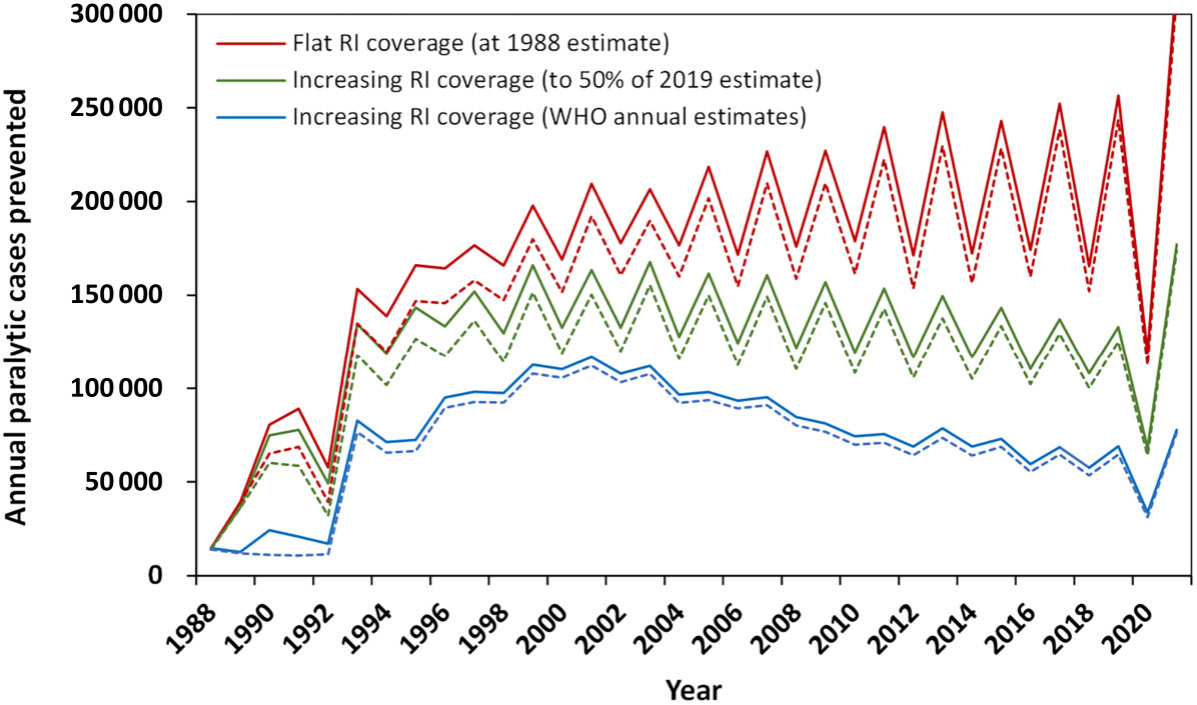
Estimated cases prevented by GPEI based on the global RC compared to a world with (1) no GPEI but with flat RI coverage at the 1988 levels (top two red curves), (2) no GPEI but with increasing RI coverage based on WHO annual estimates (bottom two blue curves), and (3) no GPEI but with moderately increasing coverage characterized by a linear increase to 50% of 2019 RI coverage (middle two green curves). For each scenario, the solid lines show the results for all 200 countries, and the accompanying dashed lines show the results for the 105 GPEI countries only. Abbreviations: GPEI, Global Polio Eradication Initiative; RC, reference case; RI, routine immunization; WHO, World Health Organization.

Visualization of the role of vaccines in prevention of paralysis (Figure 4) provides a remarkable demonstration of the substantial impact of vaccines on human health. Here, we focus on characterizing the impact of the global polio eradication since 1988, and consider either all 200 countries or only the 105 countries that received support from GPEI (GPEI countries) [3, 4]. Thus, instead of considering a counterfactual of no vaccine, we consider the counterfactual of no GPEI, with different scenarios for evolution of global RI programs in the absence of GPEI. Figure 5 shows estimated cases prevented by GPEI (from the global RC) compared to a world with (1) no GPEI but with flat RI coverage at the 1988 levels (red curves), (2) no GPEI but with increasing RI coverage based on WHO annual estimates (blue curves), and (3) no GPEI but with moderately increasing coverage characterized by a linear increase to 50% of 2019 RI coverage (green curves). In Figure 5, the solid lines show the results for all 200 countries, while the dashed lines show the results for the 105 GPEI countries only.

For any specific time period, we can sum the annual numbers to estimate the cumulative cases. For example, for 1960–1987, the results in Figure 3 sum to 12 378 619 for the global RC and 17 509 090 for no vaccine, which leads to an estimate of 5 130 471 prevented cases by vaccines as used prior to 1988. For 1988–2021, Table 2 provides the cumulative estimated paralytic polio cases (second column) for each modeled scenario (first column) for all countries (top) and the 105 GPEI countries (bottom). The RC provides the best available estimates of the actual global experience with polio (accounting for underreporting), and the other scenarios provide estimates of different counterfactual paths the world could have taken. Using the cumulative numbers for each scenario, we can then subtract the cumulative cases for the RC from the other scenarios. Comparison of no vaccine to the RC provides estimates of the impact of the existence and use of polio vaccines, whereas comparison of each no GPEI result (Figure 5) with the RC characterizes the impact of GPEI. For 1988–2021, the total number of cases prevented by GPEI range from 2.5 to 6 million depending on the counterfactual scenario. As anticipated, most of the benefits of GPEI are realized in GPEI countries, because routine childhood immunization programs in developed countries eliminated polio without GPEI. Comparison of the 3 no GPEI scenarios highlights uncertainty about the impact of the assumptions made about progress in childhood immunization programs that would have occurred without GPEI. Although, GPEI unquestionably contributed to the global advancement of childhood immunization programs in some countries [21], these programs may have increased polio immunization regardless of the global commitment to eradicate polio.

**Table 2.** Estimated Cumulative Impact of Poliovirus Immunizations Interventions for 1988 and 2021 With Different Analytical Framing Assumptions

Estimated Totals from Modeling for Specific Countries and Scenarios	Paralytic Cases	Paralytic Cases Prevented vs Global RC
All countries		
Global RC	840 793	…
No vaccine	24 854 075	24 013 282
No GPEI, flat RI coverage, fixed at 1988 estimate	6 784 532	5 943 739
No GPEI, increasing RI coverage, to 50% of 2019 estimate	5 054 183	4 213 389
No GPEI, increasing RI coverage, WHO annual estimates	3 361 430	2 520 637
GPEI countries		
Global RC	790 642	…
No vaccine	18 945 465	18 154 823
No GPEI, flat RI coverage, fixed at 1988 estimate	6 208 673	5 418 031
No GPEI, increasing RI coverage, to 50% of 2019 estimate	4 614 741	3 824 099
No GPEI, increasing RI coverage, WHO annual estimates	3 149 599	2 358 957

Abbreviations: GPEI, Global Polio Eradication Initiative; RC, reference case; RI, routine immunization; WHO, World Health Organization.

## DISCUSSION

Although polio represents an interdependent risk of international concern, each country collects and reports its own national data. Polio surveillance systems have varied substantially between countries and over time. Reporting of the available polio data began in the mid-20th century by some developed countries individually [1], and by WHO or its predecessor [23], prior to the availability of licensed vaccines. By the 1970s, WHO published annual summaries of reported cases including updated estimates for prior years. Because polio reporting began before diagnostic testing became readily available, reported cases primarily refer to paralytic cases. As such, the total number of poliovirus infections, including essentially all asymptomatic and nonparalytic infections, are not included in reported or estimated cases.

Reported cases (Figure 1) provide the true burden of disease only with a perfect disease surveillance system that (1) counts every case of paralysis that is polio, and (2) does not count cases of paralysis that are not polio. Efforts to retroactively identify missed cases have included time-consuming and labor-intensive field studies, but these efforts are generally unable to compensate for the entirety of underreported cases. Early on, GPEI recognized the need for nearly perfect surveillance so that it could identify places where poliovirus transmission was occurring, and this led to the development and support of an extensive disease surveillance system. The improvement in polio surveillance that occurred, largely due to GPEI investments, is seen by gradual convergence of the RC and reported cases over time (Figure 3 and inset).

Although we identified multiple published estimates of prevented cases (Figure 2), we failed to identify published analytical support for the widely publicized figure of 350 000 cases in 1988 (outlying red diamond symbol in Figure 2), which has become a GPEI benchmark [43–46]. Based on our search, this estimate first appeared in a 1999 publication with no reference to source or methods [33], and exceeds previous WHO estimates [31] by 100 000–150 000 cases. Notably, the same figure appears in WHO’s progress report for 1986–1988 in a different form as “it is estimated that 350 000 cases of paralytic poliomyelitis were prevented in 1988” [31], raising the possibility of misquotation in the subsequent literature. Alternatively, it is possible that the 350 000 figure is a factor-of-10 multiplication of the reported cases in 1988 (Figure 1, currently at 34 617 cases). If so, this would be based on the belief that “in 1987, only some 10% of the quarter of a million cases of poliomyelitis which were estimated to occur in that year were officially reported” [47]. Over/underestimating the burden of paralytic polio by a significant fraction could have a large impact on estimates of the health and economic benefits of GPEI since its inception in 1988.

The results presented here provide a powerful reminder of the positive global impact of vaccines on human health. The public perception of polio is often as a disease of the past, but the dynamics of polio control (Figure 3) and cases prevented (Figure 4) demonstrate that the global fight against polio spans decades of commitment to immunizations, surveillance, funding, and other activities worldwide. Figure 5 also shows that the global commitment to eradicate polio has been a remarkable success story, especially for GPEI countries in which polio resources have also improved and expanded overall health systems [48]. GPEI infrastructure plays a key role in surveillance for other infectious diseases [49] and public health responses to emerging infectious diseases (eg, severe acute respiratory syndrome [SARS], Ebola, and the COVID-19 pandemic) as well as population disruptions due to natural disasters and conflict [50].

While simple estimations of cases and cases prevented can introduce significant errors by overly simplified input assumptions, the estimates calculated using the global model also come with limitations associated with the model structure, assumptions, and available information (see details in [15]). We considered the impact of COVID-19 on global immunization dynamics [20], but acknowledge uncertainty related to the true and not yet fully observed impacts of COVID-19 pandemic on RI activities, preventive and outbreak SIAs, population mixing, and transmission dynamics. The no vaccine curves in Figure 3 and Figure 4 provides some indication of the substantial decrease in population mixing and opportunities for poliovirus transmission that occurred in 2020 as the pandemic shutdown travel, work and social activities, and restricted spread due to increased cleaning, social distancing, and mask use. As these disruptions abated in 2021, we see a substantial increase in cases for no vaccine, and for the global RC, although the resumption of immunization activities tempered the increase in RC.

Analysis of historical cases and cases prevented can provide important context for the benefits of investments made in polio eradication. By focusing on paralytic cases, our analysis undervalues prevention of substantial burdens of disability and deaths. Future studies will need to explore the expected benefits of prospective investments. Prior modeling explored both retrospective and prospective estimates of cases prevented [3, 4], but the prospective outcomes will depend on whether and when GPEI succeeds and the path taken. As polio eradication efforts continue, additional studies will also need to extend the retrospective modeling to account for changes in immunization coverage, which may depend on global recovery from the disruptions of the COVID-19 pandemic and continued investments in GPEI.

## Notes

***Author contributions.*** K. B. contributed to investigation, data visualization, and writing. D. A. K. contributed to modeling executions, data visualization, and writing. K. M. T. contributed to conceptualization of the project, data visualization, supervision, and writing.
